# Prior-Enhanced Axial TransUNet: integrating MedSAM priors and sparse region-aware attention for kidney-tumor segmentation

**DOI:** 10.3389/frai.2026.1848075

**Published:** 2026-09-30

**Authors:** Siyuan Liang, Cheng Xu, Chao Lu, Jiewen Bao, Jiangchun Wu, Xin Gu, Bin Xu

**Affiliations:** 1 Department of Urology, Shanghai Ninth People's Hospital, Shanghai Jiao Tong University School of Medicine, Shanghai, China; 2 Automation Business Division, Shanghai Baosight Software Co., Ltd., Shanghai, China; 3 Department of Gynecologic Oncology, Fudan University Shanghai Cancer Center, Shanghai, China

**Keywords:** dual-path fusion, kidney-tumor segmentation, medical image, region-aware attention, vision transformer

## Abstract

Accurate kidney and renal-tumor segmentation is challenging because lesion size, location, morphology, and boundary contrast vary substantially across abdominal CT scans. Most existing methods rely on a single form of local evidence and struggle to recover the boundaries of small lesions while maintaining global anatomical consistency. This paper proposes Prior-Enhanced Axial TransUNet (PEAT), which combines the broad medical representations of foundation models with task-specific discriminative learning. PEAT introduces three core components: Bottleneck Semantic Alignment (BSA) uses the frozen MedSAM image encoder as a training-stage feature reference and guides the semantic organization of the task network's bottleneck representation through an L1 alignment constraint; Dual-path Attention-enhanced Fusion (DAF) adaptively fuses CNN local features with MaxViT global contextual features by cascading coordinate attention and SENetV2; and Bi-granular Region-Aware Attention (BRA) achieves efficient non-local context modeling through Top-*k* window routing and patch-level sparse interaction. The MedSAM branch participates only during training, and only the task-specific network runs at inference. Under a setting in which all compared methods adopt the same preprocessing and evaluation protocol, PEAT achieves kidney and tumor Dice scores of 97.69% and 91.83% on KiTS21 and 96.80% and 92.58% on KiTS23, respectively. Across all four tasks, PEAT attains the highest Dice among the compared methods; ablation studies further show that the progressive integration of BSA, DAF, and BRA yields consistent gains, with the complete model outperforming all single and pairwise configurations. These results indicate that combining training-stage broad feature guidance with task-specific representation learning effectively improves kidney and renal-tumor segmentation and provides a viable route for the coordinated design of foundation-model priors and lightweight task networks.

## 1 Introduction

Medical image segmentation is commonly evaluated with pixel-level overlap metrics such as the Dice coefficient, but clinically useful predictions should also remain semantically coherent under noise, artifacts, low-contrast boundaries, and anatomical variation. Pixel-level supervision alone may overemphasize local textures and short-range discriminative cues, producing artifacts such as boundary discontinuities, holes, or organ adhesions (Ronneberger et al., 2015; Isensee et al., 2021). This issue is especially pronounced in joint kidney–tumor segmentation: the kidney is a relatively large organ with a recognizable global layout, whereas renal tumors may be small, multifocal, exophytic, or deeply embedded in the parenchyma, so a model must distinguish the kidney from surrounding abdominal structures while preserving the details of lesions with widely varying sizes and morphologies. Research has therefore progressively embedded optimizable high-level consistency constraints into the training paradigm: on the one hand, enhancing global context modeling to capture long-range dependencies and holistic morphological consistency; on the other hand, using structural priors such as topology preservation (Clough et al., 2022) and connectivity penalties (Shit et al., 2021) to guide the network toward stable, anatomy-aware representations at critical layers. This motivates incorporating representation-level guidance into the training objective rather than relying only on post-processing.

Vision foundation models offer broad medical representations learned from heterogeneous data, while task-specific models can benefit from abundant annotations for a focused application. MedSAM was developed using 1,570,263 medical image–mask pairs spanning 10 imaging modalities and more than 30 cancer types (Ma et al., 2024). PEAT combines this broad representation with task-specific learning: a frozen MedSAM encoder supplies a training-stage semantic reference, while the smaller segmentation network learns kidney- and tumor-discriminative features directly from KiTS annotations. SegMamba is a recent 3D whole-volume segmentation architecture based on state-space modeling (Xing et al., 2024) and is included in the quantitative comparison. The distinction between broad and task-specific representations is central to the design of this paper. MedSAM can encode a wide range of tissues and lesions, but a general-purpose representation is not necessarily optimal for every narrowly defined segmentation task. A dedicated model trained with abundant matched kidney–tumor labels can learn finer task-specific decision boundaries. Therefore, the proposed model does not use MedSAM to generate pseudo-labels or final masks. Instead, its frozen image embedding acts as a reference at the PEAT bottleneck, while the prediction pathway remains directly supervised by the KiTS annotations. This arrangement combines a large model's broad medical feature organization with a task-specific network's focused discrimination.

High-level semantic constraints address only one aspect of robust medical segmentation. In high-resolution images, a model should preserve boundary and texture cues for small structures while also using broader anatomical context to reduce false positives and implausible region assignments. Convolutional neural networks (CNNs) provide local inductive bias and hierarchical feature extraction (Ronneberger et al., 2015; Isensee et al., 2021), whereas Vision Transformers (ViTs) and related architectures facilitate contextual aggregation (Chen et al., 2021; Hatamizadeh et al., 2022). Each paradigm entails trade-offs when local ambiguity and global structural complexity coexist. Therefore, PEAT uses parallel CNN and MaxViT streams followed by attention-guided fusion. MaxViT (Tu et al., 2022) provides the window-grid attention backbone for multi-scale contextual modeling. For kidney–tumor CT, this local–global balance is important at several scales. Shallow features retain intensity transitions and small high-frequency structures; deeper features encode organ location, tumor–kidney relations, and broader abdominal context. Simply concatenating two feature streams does not determine which response is useful at each spatial position or channel. PEAT therefore performs multi-scale fusion with coordinate attention and SENetV2, so that spatially informative and channel-discriminative responses can be reweighted before entering the decoder.

Computational cost is also relevant when global modeling is applied to high-resolution medical images. Standard global self-attention has quadratic complexity in the number of tokens and can increase training time and GPU-memory demand (Choromanski et al., 2021; Zhu et al., 2023). Efficient-attention research explores sparsification, window partitioning, and routing to reduce selected token interactions. BiFormer's bilayer routing attention (Zhu et al., 2023) uses region screening followed by fine-grained aggregation. These mechanisms motivate the sparse-routing design of BRA. In medical images, relevant regions are not always spatially adjacent: separated parts of the same organ or multiple lesions may share semantics despite being located in different windows. BRA therefore selects candidate windows according to feature similarity and then performs patch-level interaction within the routed set. A parallel local-enhancement path retains short-range information that may not be selected by sparse routing. This combination is intended to preserve both non-local contextual interaction and local detail at the bottleneck.

To address these challenges, this paper proposes PEAT (Prior-Enhanced Axial TransUNet). The primary contributions are summarized as follows:

1. **Bottleneck Semantic Alignment (BSA)**: The model uses the frozen MedSAM image encoder as a fixed training-stage feature reference rather than as a mask generator. A lightweight projection aligns the PEAT bottleneck representation with that reference through an L1 loss, while Dice and cross-entropy supervision retain kidney–tumor task specificity. The MedSAM branch is frozen during training and is not part of the prediction pathway. The matched L1/L2/cosine comparison shows small Dice differences of 0.13–0.21 percentage points in favor of L1 over the strongest alternative for each task.
2. **Dual-path Encoding with Attention-enhanced Fusion (DAF)**: The model replaces the single encoder path with parallel CNN and MaxViT streams and fuses their multi-scale features using a Merge Block that cascades coordinate attention and SENetV2. The CNN path emphasizes local texture and edge information, whereas MaxViT provides contextual modeling. The fused features are used at the bottleneck and in the decoder skip connections, allowing contextual information and spatial detail to be integrated throughout reconstruction.
3. **Bi-granular Region-Aware Attention (BRA)**: BRA uses a bi-granular sparse attention design in which window-level Top-*k* routing selects relevant regions before patch-level interactions. A depthwise-convolutional local enhancement term complements the routed attention output and retains local positional information.
4. **Systematic verification of component complementarity**: On all four kidney and tumor segmentation tasks of two public multi-center benchmarks (KiTS21 and KiTS23), PEAT achieves the highest Dice among the compared methods. Systematic ablations (baseline, three individual modules, three pairwise combinations, and the complete model) further show that every pairwise combination exceeds its stronger constituent module and the complete model exceeds the strongest pairwise configuration, indicating that BSA, DAF, and BRA act at different stages of representation learning and that their gains are additive.

The remainder of this paper is organized as follows: Section 2 reviews related work; Section 3 presents the overall architecture of PEAT and the design of the BSA, DAF, and BRA components; Section 4 introduces the datasets and experimental settings and reports the quantitative, qualitative, and ablation results; Section 5 discusses the findings, limitations, and future directions; and Section 6 concludes the study.

## 2 Related work

### 2.1 Structural constraints and foundation-model priors

Medical image segmentation commonly relies on pixel-level objectives such as cross-entropy and Dice loss. These objectives optimize label agreement but do not explicitly constrain anatomy or connectivity, so discontinuities, holes, loss of fine structures, and adhesions may occur in difficult images (Ronneberger et al., 2015; Isensee et al., 2021). Clough et al. (2022) introduced a differentiable persistent-homology loss for connected components and topological voids. Shit et al. (2021) proposed centerline Dice (clDice) to encourage connectivity in tubular structures. These studies motivate the use of optimizable high-level constraints as complements to pixel-wise fitting. Output-level structural constraints and representation-level guidance act at different stages. Topological or centerline losses explicitly penalize properties of the predicted mask and are particularly appropriate when the desired topology is known. Feature guidance instead influences how the encoder organizes image content before the final classifier. It does not require a hand-crafted kidney or tumor template and can be combined with the ordinary segmentation objective. PEAT follows the latter strategy because the morphology and number of renal tumors vary substantially across patients.

The advent of vision foundation models (VFMs), notably the Segment Anything Model (SAM), has motivated feature reuse in medical image analysis. SAM was pretrained at billion-mask scale (Kirillov et al., 2023), while MedSAM adapted the framework using 1,570,263 medical image–mask pairs across diverse structures, lesions, and modalities (Ma et al., 2024). MedSAM provides broadly useful medical visual features, whereas dedicated models can learn stronger task-specific discrimination from matched annotations. PEAT combines these complementary properties by retaining a KiTS-trained segmentation pathway and using the frozen MedSAM image embedding as an auxiliary feature-space reference during training. Knowledge-transfer literature describes logit-, attention-, and feature-level alignment using L1/L2 distance, cosine similarity, or KL divergence (Gou et al., 2021; Wang and Yoon, 2022). These approaches motivate the feature-alignment strategy used in BSA. Feature-level transfer is well suited to this setting because the two networks do not share the same decoder or output mechanism. Aligning an intermediate embedding allows the PEAT encoder to use MedSAM's medical representation without requiring prompt simulation or agreement between two final masks. The frozen reference also provides a stable target during optimization, while all task-dependent prediction parameters remain trainable under the KiTS labels. Further support comes from self-supervised representation learning: frameworks such as DINO (Caron et al., 2021), MAE (He et al., 2022), BEiT (Bao et al., 2022), and iBOT (Zhou et al., 2022) demonstrate that high-level semantic structures can emerge without labels and transfer to downstream tasks. This supports investigating representational consistency as an auxiliary training signal.

### 2.2 Hybrid encoding and multi-scale feature fusion

Medical segmentation benefits from features that retain local detail while incorporating broader context. Boundary textures support contour delineation and small-lesion recognition, whereas cross-regional context can help resolve ambiguity and suppress false positives. CNNs provide local inductive bias, translation equivariance, and hierarchical feature extraction (Ronneberger et al., 2015; Isensee et al., 2021), but explicit interaction between distant regions can remain limited. Vision Transformers use self-attention for contextual interaction. TransUNet (Chen et al., 2021) incorporated ViT features into a U-Net-style architecture for 2D segmentation, and UNETR (Hatamizadeh et al., 2022) extended Transformer encoding to 3D segmentation. The *O*(*N*^2^) complexity of dense self-attention motivates hierarchical and hybrid architectures that balance representation and computation.

Dual-path and multi-branch designs use CNN features for local detail and Transformer features for context (Tu et al., 2022; Zhu et al., 2023). Their performance depends on how the branches are fused. Coordinate Attention (Hou et al., 2021) encodes directional position information in channel weights. Squeeze-and-Excitation networks perform channel recalibration (Hu et al., 2018), and SENetV2 extends this idea with aggregated multi-branch dense transformations (Narayanan, 2023). In medical segmentation, lightweight attention, and multi-scale fusion support adaptive selection of local and contextual features. Fusion is required at multiple resolutions because the relative value of local and contextual cues changes through the encoder. At shallow levels, high-resolution CNN responses are useful for contour recovery; at deeper levels, Transformer responses carry wider contextual relationships. The Merge Block first maps the two streams to a common channel space, then applies position-aware and channel-aware reweighting. These enhanced features are supplied through skip connections rather than fusing the two paths only once at the bottleneck.

### 2.3 Efficient attention mechanisms

Global information can help resolve local ambiguities and suppress false positives in high-resolution medical images. However, the *O*(*N*^2^) time and space complexity of standard self-attention increases computation and memory demand as the number of tokens grows (Choromanski et al., 2021; Zhu et al., 2023). Efficient global modeling therefore seeks to retain useful cross-region interactions while keeping computational cost manageable.

Efficient attention methods include approximate computation, structured interaction, and token reduction. Performer (Choromanski et al., 2021) uses random features for linear attention; Linformer (Wang et al., 2020) uses low-rank projections; and Nyströmformer (Xiong et al., 2021) uses Nyström approximation. Swin Transformer (Liu et al., 2021) propagates information through local and shifted windows, whereas BiFormer (Zhu et al., 2023) first selects relevant regions and then performs fine-grained aggregation within the routed set. ToMe (Bolya et al., 2023) reduces sequence length through token merging. Operator-level optimization provides another route: FlashAttention (Dao et al., 2022) uses IO-aware exact attention to reduce memory traffic. Together, these methods provide alternative ways to balance contextual modeling and computational cost (Liu et al., 2021; Choromanski et al., 2021; Wang et al., 2020; Xiong et al., 2021; Zhu et al., 2023; Bolya et al., 2023; Dao et al., 2022). These approaches make different compromises. Approximation changes the attention computation, token reduction shortens the sequence, and fixed windows constrain interaction by geometry. Content-aware routing instead retains patch-level attention while restricting the candidate regions for each query window. This property is attractive for abdominal CT, where two semantically related regions may be separated by background and where a fixed local window may not contain all relevant context. BRA adapts this routing principle to the PEAT bottleneck and combines it with local enhancement.

## 3 Materials and methods

### 3.1 Overall architecture overview

As shown in Figure 1, PEAT is a hybrid encoder-decoder architecture that combines an external training-stage semantic reference, dual-path feature extraction, and sparse region-aware attention.

**Figure 1:**
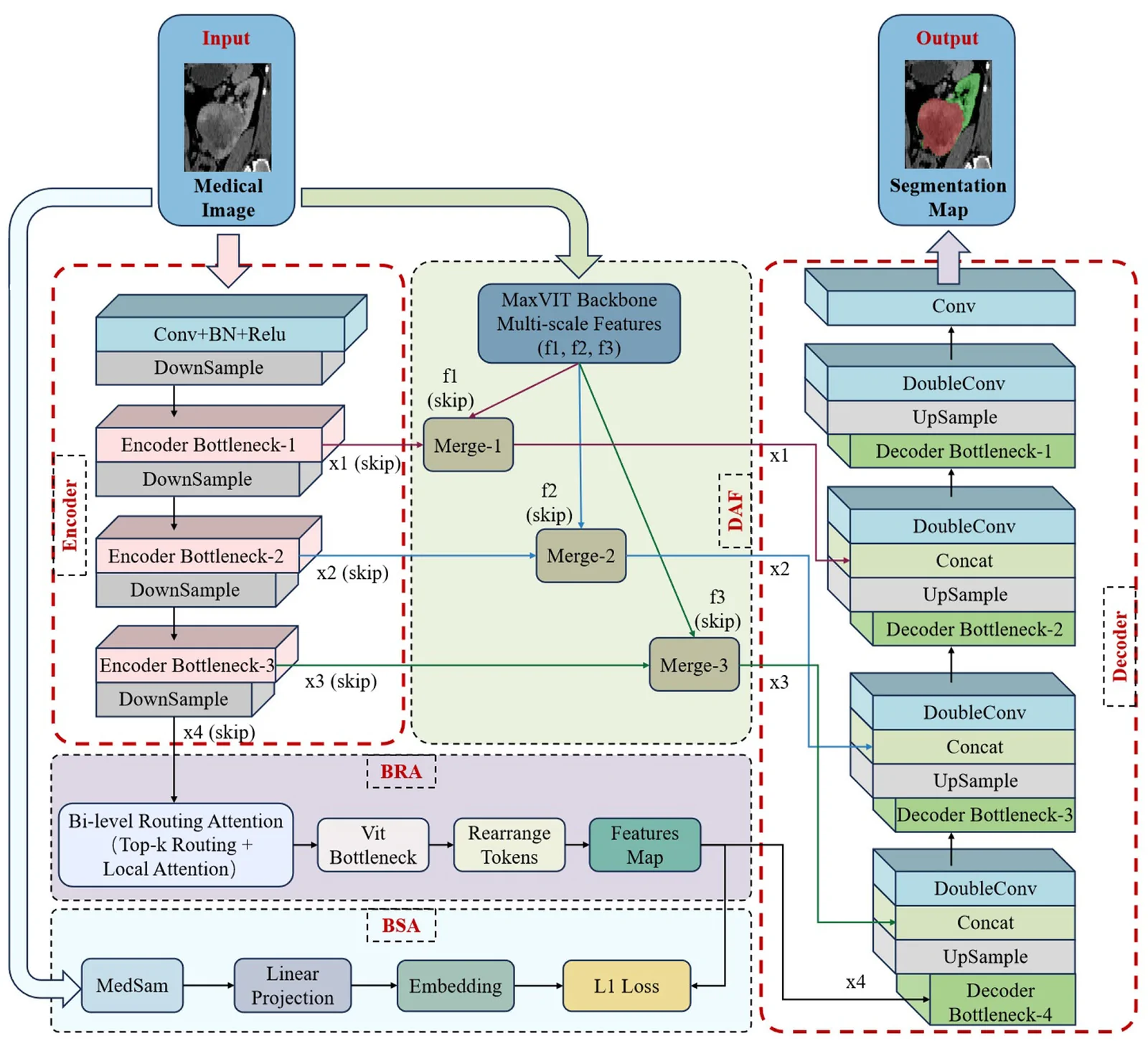
Overall architecture of Prior-Enhanced Axial TransUNet (PEAT). A ResNet-50 CNN branch extracts local features and a MaxViT branch extracts contextual features. DAF merges the two streams at multiple encoder levels, BRA performs routed interaction at the bottleneck, and the decoder produces the kidney–tumor segmentation map. The frozen MedSAM branch supplies the BSA training reference and is not used during inference.

The architecture separates representation guidance from task prediction. The ResNet-50 and MaxViT branches extract trainable task features from the same axial CT slice. Their multi-scale outputs are fused by DAF, and the deepest fused feature is processed by BRA before decoding. In parallel, the frozen MedSAM image encoder generates the feature target used by BSA. Gradients from the alignment term update the PEAT projection and segmentation network but not MedSAM. The decoder receives only PEAT features; therefore, the segmentation output is produced by the task network rather than by the MedSAM prompt encoder or mask decoder.

Specifically, given an input medical image **X**∈ℝ^*H*×*W*×*C*^, where *C* denotes the number of input channels, PEAT processes it in parallel through three branches:

1. **Backbone CNN feature extraction branch**: An ImageNet-pretrained ResNet-50 network (with parameters denoted as θ_cnn_) is employed to perform four-level downsampling on **X**, producing a local feature pyramid:
  $${\left\{F_{l}\right\}}_{l=1}^{4}=\text{CNN}\left(X\right),\;F_{l}\in {\mathbb{R}}^{\frac{H}{2^{l}}\times \frac{W}{2^{l}}\times C_{l}},$$
  where *C*_*l*_ denotes the channel dimension at encoder level *l*.
2. **Parallel MaxViT feature extraction branch**: The same input **X** is simultaneously fed into a MaxViT encoder (with parameters θ_mv_) to capture long-range and multi-scale contextual dependencies, yielding global features ${\left\{f_{l}\right\}}_{l=1}^{4}$.
3. **MedSAM feature-reference branch**: **X** is also passed through the frozen MedSAM image encoder to generate a broad medical embedding **E**_MedSAM_. This embedding is used only as an auxiliary training reference; the KiTS-specific segmentation prediction remains the responsibility of the task network.

During the encoding stage, PEAT incorporates a Dual-path Encoding with Attention-enhanced Fusion (DAF) module. At each scale level *l*, DAF feeds the local features **F**_*l*_ from the CNN pathway and the contextual features *f*_*l*_ from the MaxViT pathway into a Merge Block. This block sequentially integrates Coordinate Attention (CoordAtt), convolutional transformation, and SENetV2 channel recalibration to achieve adaptive weighting across spatial and channel dimensions, ultimately producing enhanced features:

$$F_{\text{enh}}^{l}=\text{Merge}\left(F_{l},f_{l}\right).$$

The deepest enhanced feature $F_{\text{enh}}^{4}$ serves as the input to the bottleneck layer and is directly fed into a Transformer module based on Bi-granular Region-Aware Attention (BRA). This module takes a 2D feature map as both input and output, and its computation can be formalized as:

$$F_{\text{bottleneck}}=\text{BRA}\left(F_{\text{enh}}^{4}\right),$$

where BRA employs a Top-*k* routing strategy to perform coarse-grained region selection at the window level, followed by fine-grained patch-level interactions within the selected regions. The routing design reduces dense token interactions relative to standard global attention. The resulting feature map, denoted as **F**_bottleneck_, is then used as the initial input to the decoder.

In the decoding stage, PEAT adopts a U-Net-style skip-connection architecture, but replaces the original CNN features with the enhanced features $\left\{F_{\text{enh}}^{3},F_{\text{enh}}^{2},andF_{\text{enh}}^{1}\right\}$ produced by DAF. This allows stronger semantic information to be injected during spatial detail recovery. The decoder consists of multiple upsampling blocks, each comprising a DoubleConv operation, feature concatenation (Concat), and upsampling (UpSample), progressively reconstructing a high-resolution segmentation map. Ultimately, the decoder outputs the predicted segmentation $\hat{Y}$.

This information flow assigns different responsibilities to the three components. DAF determines how local and contextual encoder features are combined at each scale; BRA controls cross-region interaction in the deepest representation; and BSA shapes that representation through an auxiliary objective during training. The components are therefore connected sequentially in the task pathway but intervene at distinct operations. Their individual, pairwise, and complete configurations are evaluated in the ablation study.

The entire network is optimized end-to-end using two loss terms applied at distinct locations. The first loss operates at the output layer to supervise pixel-wise segmentation accuracy and is composed of Dice loss and cross-entropy loss:

$$L_{\text{dice}}=1-\frac{2\sum \hat{Y}⊙Y}{\sum \hat{Y}+\sum Y},\;L_{\text{ce}}=-\sum Y\log\left(\hat{Y}\right),$$

where **Y** denotes the ground-truth label and ⊙ represents element-wise multiplication.

The second loss is applied at the bottleneck layer—namely, the Bottleneck Semantic Alignment (BSA) mechanism—to guide representation learning:

$$L_{\text{align}}=\frac{1}{hwD_{\text{sam}}}{\left\|P\left(F_{\text{bottleneck}}\right)-E_{\text{MedSAM}}\right\|}_{1},$$

where P(·) denotes a linear projection used to align feature dimensions. This loss encourages the PEAT bottleneck features to approach the frozen MedSAM embedding.

Consequently, the total loss function is formulated as a weighted sum of the two components:

$$L_{\text{total}}=L_{\text{dice}}+L_{\text{ce}}+\lambda L_{\text{align}},$$

where λ is a hyperparameter that balances segmentation accuracy and semantic alignment strength; this work sets λ = 1 (see Section 4.2).

### 3.2 Bottleneck Semantic Alignment (BSA)

In medical image segmentation, broad semantic representations can complement the local texture and edge features learned from a task-specific dataset. PEAT retains direct Dice and cross-entropy supervision from the KiTS ground truth, while MedSAM supplies a fixed image-level feature reference during training.

The Bottleneck Semantic Alignment (BSA) mechanism implements this large-model/small-model combination. It uses the frozen MedSAM image-encoder embedding as an external semantic reference and applies an L1 constraint to the projected PEAT bottleneck representation. The objective is to transfer broad medical feature organization while preserving the task-specific discrimination learned from KiTS. BSA operates at the feature level, and the KiTS-trained decoder produces the segmentation prediction.

The bottleneck is selected as the alignment site because it contains the most compressed, semantically aggregated task representation before spatial reconstruction. Aligning at this level avoids imposing direct agreement between low-level texture responses from architectures with different encoders. It also leaves the decoder free to reconstruct kidney and tumor boundaries using the DAF-enhanced skip features. Thus, BSA constrains the organization of high-level features without replacing the ordinary pixel-wise supervision.

Specifically, given an input image **X**∈ℝ^*H*×*W*×*C*^, the frozen MedSAM image encoder (denoted Enc_sam_, with parameters fixed) produces the corresponding image embedding:

$$E_{\text{MedSAM}}={\mathrm{Enc}}_{\text{sam}}\left(X\right)\in {\mathbb{R}}^{h\times w\times D_{\text{sam}}}.$$

The embedding has channel dimension *D*_sam_, and its spatial resolution is aligned with that of the PEAT bottleneck layer.

BSA uses only the frozen MedSAM image encoder, without prompt input, mask-decoder invocation, or an interactive step. MedSAM contributes a broad representation learned from heterogeneous medical data, whereas the PEAT segmentation pathway is optimized for the specific kidney-tumor labels.

This choice also addresses the difference between general recognition and specialized segmentation. A foundation model may encode diverse organs and lesions, yet a dedicated network trained on a large set of matched labels can be more sensitive to the appearance distribution and class definitions of one task. BSA therefore treats MedSAM as a representation reference, not as a teacher whose final prediction must be copied. Direct KiTS supervision remains the source of the kidney and tumor decision boundaries.

Due to the channel dimension mismatch (*D*≠*D*_sam_), a lightweight linear projection $P:{\mathbb{R}}^{D}\to {\mathbb{R}}^{D_{\text{sam}}}$ is introduced at the end of the bottleneck layer:

$$F_{\text{proj}}=P\left(F_{\text{bottleneck}}\right)=F_{\text{bottleneck}}W_{p}+b_{p},$$

where $W_{p}\in {\mathbb{R}}^{D\times D_{\text{sam}}}$ and $b_{p}\in {\mathbb{R}}^{D_{\text{sam}}}$; the multiplication acts on the last (channel) dimension and the bias is broadcast over the spatial dimensions. Here, **F**_bottleneck_ denotes the bottleneck feature map produced by the BRA module. This projection is used exclusively for alignment-loss computation.

Based on this alignment, the BSA loss is defined as the element-wise L1 norm:

$$L_{\text{align}}=\frac{1}{hwD_{\text{sam}}}{\left\|F_{\text{proj}}-E_{\text{MedSAM}}\right\|}_{1}.$$

This loss is jointly optimized with the segmentation task. Let $L_{\text{dice}}$ and $L_{\text{ce}}$ denote the Dice loss and cross-entropy loss, respectively. The overall training objective is then formulated as:

$$\begin{array}{cc} \underset{\theta ,\psi }{\min}\;{𝔼}_{\left(X,Y\right)\sim D}\; & L_{\text{dice}}\left(\hat{Y},Y\right)+L_{\text{ce}}\left(\hat{Y},Y\right)\\ & +\lambda L_{\text{align}}\left(X\right)\;, \end{array}$$

where θ denotes the trainable task-network parameters, ψ denotes the projection parameters, λ>0 is the alignment weight, and $\hat{Y}$ represents the model's prediction. The alignment term depends on the input image rather than directly on the ground-truth label **Y**. During training, the MedSAM encoder is frozen and its output is treated as a constant.

From the perspective of representation learning, BSA minimizes the feature discrepancy between the projected PEAT bottleneck and the medical embedding produced by the frozen encoder for the same input. Dice and cross-entropy supervision simultaneously preserve kidney- and tumor-specific discrimination.

During back-propagation, the MedSAM embedding is detached from parameter updates. The alignment gradient is propagated through the projection and the PEAT encoder, while the segmentation gradient is propagated through the entire task network. The two objectives meet at the bottleneck: one encourages compatibility with the broad medical feature reference, and the other enforces agreement with the kidney–tumor labels. At inference, only the task pathway is evaluated.

L1 was selected because it gives a constant-magnitude subgradient away from zero and is less sensitive to large residuals than the squared L2 distance; the matched comparison (Section 4.5) shows that L1 attains the highest Dice on all four tasks, with margins of only 0.13–0.21 percentage points over the strongest alternative.

### 3.3 Dual-path Encoding with Attention-enhanced Fusion (DAF)

TransUNet combines convolutional features with a Transformer operating on a high-level feature map. PEAT instead uses a dual-stream design in which CNN and MaxViT features are produced at multiple scales before fusion.

Accordingly, the model uses a Dual-path Encoding with Attention-enhanced Fusion (DAF) architecture. As illustrated in Figure 2, a CNN backbone extracts local features while a MaxViT encoder captures global context; the Merge Block fuses their multi-scale outputs. Both encoders receive the same input image **X**.

**Figure 2:**
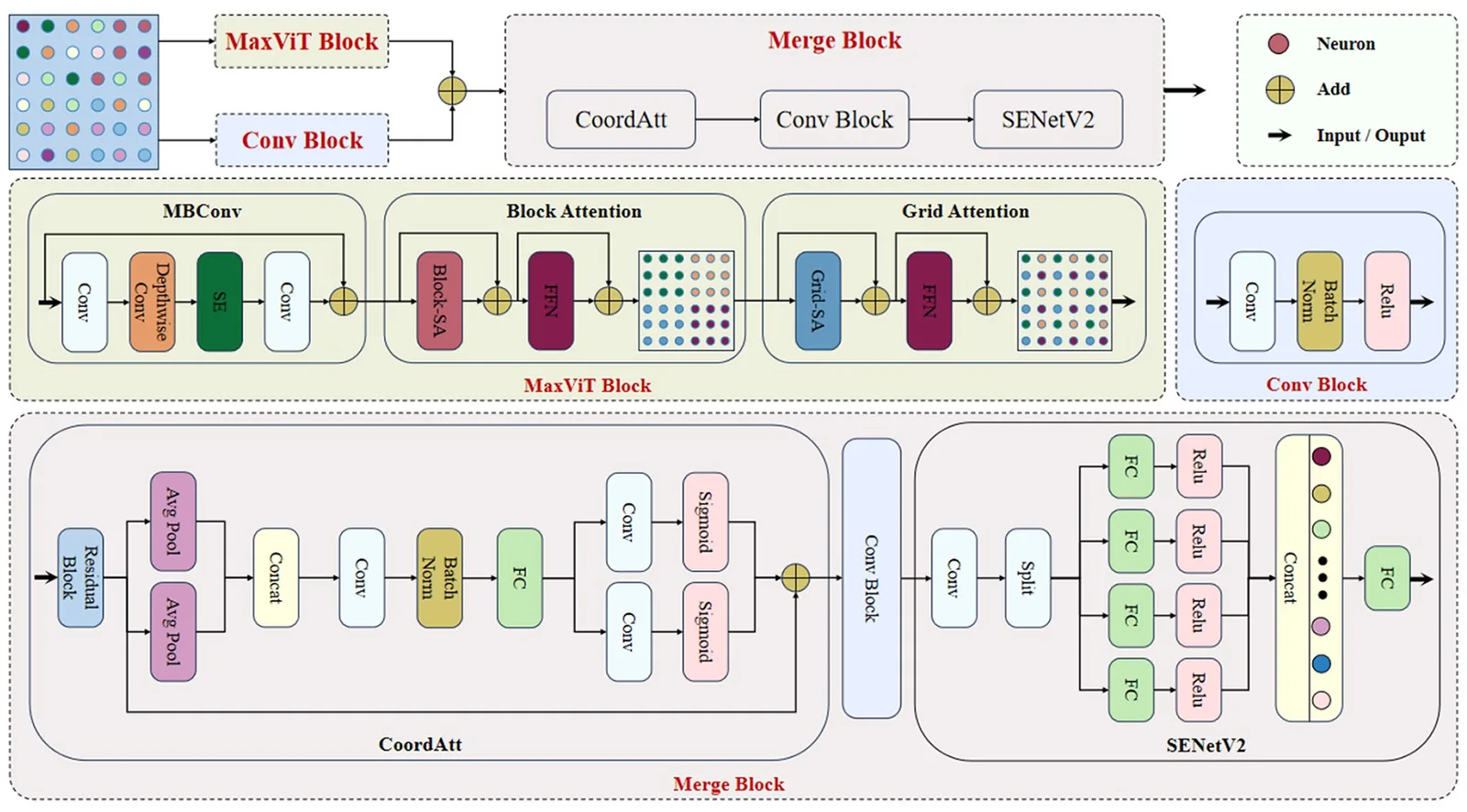
Dual-path encoding and attention-enhanced fusion. The CNN and MaxViT branches process the same input in parallel. At each scale, the Merge Block combines Coordinate Attention, convolutional refinement, and SENetV2 channel recalibration before passing the fused feature to the corresponding decoder level.

The parallel arrangement differs from inserting a Transformer only after the final CNN stage. Both streams preserve a hierarchy of features, allowing fusion to occur before each corresponding decoder stage. This is useful for renal CT because a small lesion may depend strongly on shallow intensity and edge cues, whereas a large or irregular tumor may require deeper contextual features to separate it from adjacent tissue.

Specifically, given an input image **X**∈ℝ^*H*×*W*×*C*^, DAF constructs two fully parallel feature extraction pathways. The first pathway is the CNN branch, where **X** is fed into an ImageNet-pretrained ResNet-50 backbone network to generate a four-level local feature pyramid:

$${\left\{F_{l}\right\}}_{l=1}^{4}=\text{CNN}\left(X\right),\;F_{l}\in {\mathbb{R}}^{\frac{H}{2^{l}}\times \frac{W}{2^{l}}\times C_{l}},$$

where *C*_*l*_ denotes the channel dimension at encoder level *l*. This pathway excels at preserving high-frequency textures and fine boundaries, providing critical support for the detection of small lesions. The second pathway is the MaxViT branch, in which the same input **X** is simultaneously fed into a MaxViT encoder to produce the corresponding four-level global features:

$${\left\{f_{l}\right\}}_{l=1}^{4}=\text{MaxViT}\left(X\right),\;f_{l}\in {\mathbb{R}}^{\frac{H}{2^{l}}\times \frac{W}{2^{l}}\times D_{l}}.$$

MaxViT uses a window-grid attention mechanism for global-context modeling. The parallel dual-path design produces separate local and contextual feature streams before fusion. DAF operates in the encoder and supplies fused multi-scale features; BRA subsequently refines bottleneck interactions, while BSA adds an auxiliary training constraint at the bottleneck. Because these modules act on different functional aspects rather than competing for the same output role, their design is complementary. The pairwise and full-model ablations in Section 4 directly verify that their progressive integration yields further performance gains.

To fuse these two heterogeneous feature sets, the model introduces a Merge Block at each scale level *l*. This module first projects **F**_*l*_ and *f*_*l*_ into a shared channel dimension *C* (default *C* = 512) using 1 × 1 convolutions, then concatenates the results along the channel dimension to form $F_{\text{cat}}\in {\mathbb{R}}^{h\times w\times 2C}$. Subsequently, it applies Coordinate Attention (CoordAtt), followed by a lightweight convolutional block and a SENetV2 module, to perform adaptive feature enhancement.

The 1 × 1 projections make the heterogeneous branch outputs compatible without changing their spatial resolution. Concatenation preserves information from both streams, after which the attention cascade performs selection rather than treating all responses equally. Coordinate Attention first introduces directional spatial modulation, and SENetV2 subsequently recalibrates the resulting channels. The ordering therefore reflects a spatial-to-channel refinement process within each Merge Block.

The incorporation of CoordAtt stems from the prevalent strong directional anatomical structures in medical images, such as the vertical orientation of the spine, the horizontal extension of intestinal loops, or the tree-like branching of vasculature. To explicitly model this direction-aware property, CoordAtt first applies global average pooling to the input feature **U**∈ℝ^*h*×*w*×*C*^ along the horizontal and vertical spatial dimensions, respectively:

$$z_{h}\left(i,c\right)=\frac{1}{w}\overset{w}{\underset{j=1}{\sum }}U\left(i,j,c\right),\;z_{w}\left(j,c\right)=\frac{1}{h}\overset{h}{\underset{i=1}{\sum }}U\left(i,j,c\right),$$

yielding two directional descriptors $z_{h}\in {\mathbb{R}}^{h\times 1\times C}$ and $z_{w}\in {\mathbb{R}}^{1\times w\times C}$. They are concatenated along the spatial dimension and transformed by a shared dimensionality-reduction layer. The transformed descriptor is then split into horizontal and vertical components, **f**_*h*_ and **f**_*w*_, which are expanded separately to generate the directional attention weights:

$$\begin{array}{cccc} f & =\delta \left(\left[z_{h};z_{w}\right]W_{1}\right), & \left[f_{h};f_{w}\right] & =\mathrm{Split}\left(f\right),\\ a_{h} & =\sigma \left(f_{h}W_{h}\right), & a_{w} & =\sigma \left(f_{w}W_{w}\right), \end{array}$$

where $W_{1}\in {\mathbb{R}}^{C\times \left(C/r\right)}$ is the shared reduction transform, $W_{h},W_{w}\in {\mathbb{R}}^{\left(C/r\right)\times C}$ are the direction-specific expansion transforms, *r* denotes the reduction ratio, and δ(·) and σ(·) represent the ReLU and sigmoid activation functions, respectively. The transforms act on the last (channel) dimension. Finally, the output features are modulated via element-wise multiplication:

$$U_{\text{out}}=U⊙\text{Expand}\left(a_{h}\right)⊙\text{Expand}\left(a_{w}\right),$$

where ⊙ denotes element-wise multiplication and Expand(·) represents broadcasting. The row- and column-wise weights provide positional modulation.

Following CoordAtt, a lightweight convolutional block is employed to further refine the features and introduce nonlinearity. Subsequently, a SENetV2 module (Narayanan, 2023) is applied to model dynamic inter-channel dependencies. Given its input **V**∈ℝ^*h*×*w*×*C*^, SENetV2 first generates a channel-wise descriptor via global average pooling:

$$s\left(c\right)=\frac{1}{hw}\overset{h}{\underset{i=1}{\sum }}\overset{w}{\underset{j=1}{\sum }}V\left(i,j,c\right).$$

In contrast to the single-branch structure of the standard SE Block, the implemented SENetV2 block splits **s** into *B* subsets and applies an independent fully connected transform and ReLU activation to each subset **s**_*i*_:

$$h_{i}=\delta \left(s_{i}W_{i}+b_{i}\right),\;i=1,\ldots ,B.$$

The branch outputs are concatenated and passed through a final fully connected layer and sigmoid gate to form the complete channel-wise weight vector:

$$e=\sigma \left(\left[h_{1};\ldots ;h_{B}\right]W_{o}+b_{o}\right)\in {\mathbb{R}}^{C}.$$

Here, *B* is the number of parallel subsets and all fully connected transforms act on the channel dimension. The final output is then given by:

$$V_{\text{out}}=V⊙\text{Expand}\left(e\right).$$

This multi-branch design models channel-wise interactions and reweights the fused CNN and MaxViT responses.

Because the same Merge operation is applied at the four encoder levels, the decoder receives a consistent hierarchy of enhanced skip features. Shallow enhanced features contribute spatial detail during upsampling, while the deepest enhanced feature carries the most aggregated local–global representation into BRA. DAF thus affects both the bottleneck input and the information recovered through skip connections.

CoordAtt applies spatially informed weighting, whereas SENetV2 applies channel weighting. Their cascade provides both forms of modulation within the Merge Block, and the contribution of DAF is evaluated in the single-module and combination ablations in Section 4.

The output of the entire Merge Block is thus the enhanced feature at that scale level:

$$F_{\text{enh}}^{l}=\text{SENetV2}\left(\text{Conv Block}\left(\text{CoordAtt}\left(F_{\text{cat}}\right)\right)\right).$$

DAF provides multi-scale fused features: shallow levels retain local-detail information and deep levels provide inputs to the bottleneck. The deepest feature $F_{\text{enh}}^{4}$ is passed to BRA.

### 3.4 Bi-granular Region-Aware Attention (BRA)

Standard global self-attention computes pairwise interactions among all tokens and therefore has quadratic complexity *O*(*N*^2^), where *N* = *HW*/*P*^2^ and *P* denotes the token patch size. BRA uses sparse routing to reduce the number of token interactions considered at the bottleneck.

The purpose of routing is not to discard global context altogether, but to make the candidate set content dependent. Each query window first obtains a compact regional descriptor. Similarity among these descriptors determines which windows may exchange detailed information. Patch-level attention is then evaluated inside the selected candidate regions, permitting non-adjacent but semantically related regions to interact.

Therefore, this model employs Bi-granular Region-Aware Attention (BRA) at the bottleneck layer to reduce the interaction overhead. Figure 3 illustrates its two-stage sparse-routing design.

**Figure 3:**
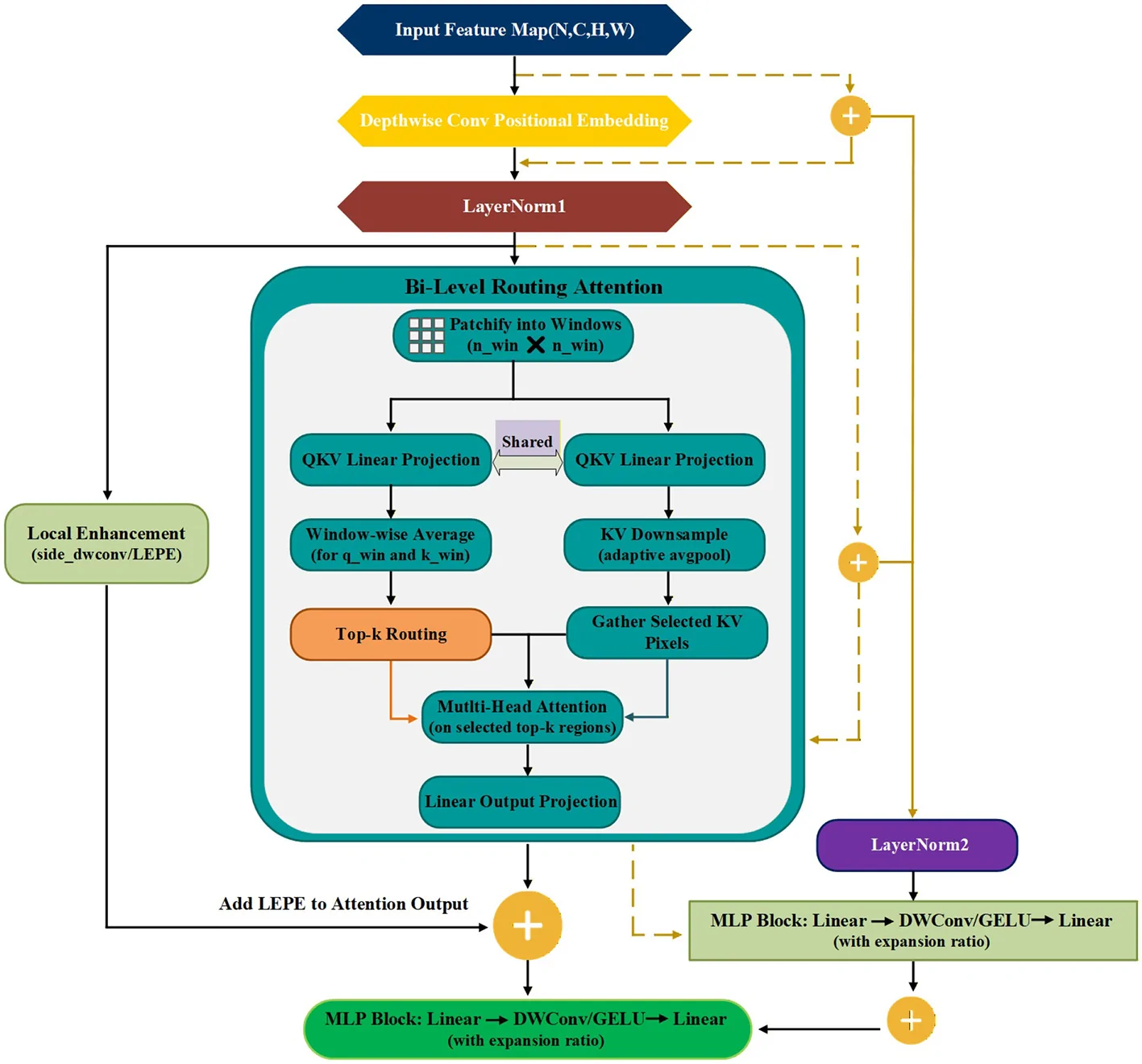
Bi-granular Region-Aware Attention (BRA). Window descriptors guide Top-*k* region routing, selected key–value tokens support fine-grained multi-head attention, and a local positional enhancement branch preserves spatial detail. Residual connections and the MLP block produce the updated bottleneck representation.

Let the input to the BRA module be the feature map $F_{\text{in}}\in {\mathbb{R}}^{h\times w\times D}$ from the encoder, where *h* = *H*/16, *w* = *W*/16, and *D* denotes the channel dimension. The entire BRA module is composed of *L* identical stacked layers. This paper first describes the computational flow of a single layer.

Given the input feature map **F**∈ℝ^*h*×*w*×*D*^ of the current layer, a depthwise-convolutional positional embedding is first added through a residual connection. The result is normalized by LayerNorm and flattened into sequence form:

$$\begin{array}{cc} F_{\text{pos}} & =F+{\mathrm{DWConv}}_{\text{pos}}\left(F\right),\\ Z & =\mathrm{Flatten}\left(\mathrm{LayerNorm}\left(F_{\text{pos}}\right)\right)\in {\mathbb{R}}^{N\times D},\;N=hw. \end{array}$$

Learned projections then produce query, key, and value tokens, **Q** = **ZW**^*Q*^, **K** = **ZW**^*K*^, and **V** = **ZW**^*V*^. Subsequently, the Bi-Level Routing Attention is computed in two stages.

**Stage 1**: Top-*k* Routing (coarse-grained region selection). The feature map is partitioned into *n*_win_×*n*_win_ non-overlapping regions $\left\{W_{1},\ldots ,W_{M}\right\}$, where $M=n_{\text{win}}^{2}$. For each region $W_{i}$, query, and key descriptors are computed by averaging their projected tokens:

$$q_{i}^{r}=\frac{1}{\left|W_{i}\right|}\underset{p\in W_{i}}{\sum }q_{p},\;k_{i}^{r}=\frac{1}{\left|W_{i}\right|}\underset{p\in W_{i}}{\sum }k_{p}.$$

Next, the region-affinity matrix **S**^win^∈ℝ^*M*×*M*^ is computed from the projected descriptors:

$$S_{ij}^{\text{win}}=\frac{{\left(q_{i}^{r}\right)}^{⊤}k_{j}^{r}}{\sqrt{d_{r}}},$$

where *d*_*r*_ is the routing-descriptor dimension.

For each query window $W_{i}$, the Top-*k* most relevant windows (including itself) are retained to form the high-relevance window set $N_{i}$.

Including the query window ensures that local within-window interaction is retained, whereas the remaining routed windows provide non-local candidates. The value of *k* controls the breadth of the candidate context: a smaller value of *k* increases sparsity, whereas a larger value tends toward denser interaction. In the reported configuration, the same routing setting is used throughout the stacked BRA layers.

**Stage 2**: Patch-level fine-grained interaction. For a query patch $z_{p}\in W_{i}$, the key–value tokens in each routed window are adaptively average-pooled and then gathered, as shown in Figure 3. Let $R_{i}$ denote the resulting routed token set; it includes tokens from the query window. For one attention head, the attention weights are defined as:

$$\begin{array}{cc} \alpha_{pq} & =\frac{\exp\left(q_{p}^{⊤}k_{q}/\sqrt{d_{h}}\right)}{\underset{r\in R_{i}}{\sum }\exp\left(q_{p}^{⊤}k_{r}/\sqrt{d_{h}}\right)},\;q\in R_{i}, \end{array}$$

where *d*_*h*_ is the per-head query/key dimension. The attention output for this patch is:

$${\hat{z}}_{p}=\underset{q\in R_{i}}{\sum }\alpha_{pq}v_{q}.$$

The outputs of all patches are then reassembled into the feature map:

$$\hat{F}=\text{Reshape}\left({\left[{\hat{z}}_{1},\ldots ,{\hat{z}}_{N}\right]}^{⊤}\right)\in {\mathbb{R}}^{h\times w\times D}.$$

This design is motivated by the spatial coherence of many anatomical structures and lesions. Window-level descriptors summarize regional content, while Top-*k* routing directs patch-level attention toward semantically related regions and their context. Swin Transformer propagates information across neighboring windows through shifted-window operations, whereas BRA selects candidate windows according to learned semantic relevance; the distinction therefore lies in the routing criterion rather than an absolute inability of window-based attention to exchange cross-window information. The two-stage design first selects regions and then performs finer interactions within the routed set, focusing computation on relevant regions.

For kidney–tumor segmentation, content-based routing can associate spatially separated areas with similar organ or lesion features while avoiding uniform interaction with every background window. At the same time, regional descriptors are coarser than individual patches and may omit short-range detail. The LEPE branch is therefore added in parallel to preserve local spatial evidence.

To compensate for potential loss of local spatial details caused by sparse attention, BRA incorporates a Local Enhancement (LEPE) module. This module applies a side depthwise convolution to the position-enhanced feature to generate a local term:

$$\Delta F={\mathrm{DWConv}}_{\text{LEPE}}\left(F_{\text{pos}}\right)\in {\mathbb{R}}^{h\times w\times D}.$$

The routed attention update, local enhancement, and position-enhanced residual are combined as follows:

$$F_{\text{attn}}=F_{\text{pos}}+\hat{F}+\Delta F.$$

After the attention computation, the layer is updated through an MLP block containing linear projection, depthwise convolution, GELU activation, and a final linear projection, followed by a residual connection:

$$F^{\text{out}}=\text{MLP}\left(\text{LayerNorm}\left(F_{\text{attn}}\right)\right)+F_{\text{attn}}.$$

Let $F^{\left(0\right)}=F_{\text{in}}$, and recursively apply the above process *L* times, that is, **F**^(*l*)^ = BRA-Layer(**F**^(*l*−1)^) for *l* = 1, …, *L*. The final output of the BRA module—the bottleneck feature fed into the decoder—can then be expressed as:

$$F_{\text{bottleneck}}=F^{\left(L\right)}.$$

Overall, BRA combines Top-*k* region routing with a local enhancement path, and its contribution is evaluated through the reported single-module, pairwise, and complete-model ablations.

Stacking the BRA layers repeatedly updates both routed contextual features and locally enhanced features. The resulting bottleneck is passed to the decoder and is also projected for BSA. Consequently, BRA participates in the task prediction pathway, while BSA evaluates the semantic organization of its output during training.

## 4 Experiments and results

### 4.1 Datasets

The proposed model is evaluated on the publicly available KiTS21 and KiTS23 kidney and renal-tumor segmentation benchmarks (Kidney and Kidney Tumor Segmentation Challenge, 2021, 2023). These multi-center, multi-scanner datasets contain contrast-enhanced abdominal CT volumes with expert annotations. KiTS21 provides 300 cases, while the KiTS23 challenge comprises 489 training cases and 110 held-out test cases. The present experiments use axial CT slices for kidney and tumor segmentation.

These datasets present significant technical challenges. Renal boundaries may be ambiguous because of adjacent structures (e.g., liver or spleen) or pathological alterations. Tumors exhibit substantial heterogeneity in morphology, size, and location, ranging from small focal lesions to large masses occupying renal units. Noise, metal artifacts, and low-contrast regions further complicate segmentation.

The two tasks also emphasize different error patterns. Kidney segmentation is dominated by the extent of a comparatively large anatomical structure, but the contour may be interrupted by tumor invasion or confused with nearby organs of similar attenuation. Tumor segmentation involves a smaller and more heterogeneous foreground, making missed regions, fragmented predictions, and false positive islands more influential. Reporting kidney and tumor results separately therefore provides a clearer view than collapsing both labels into a single foreground class.

This paper compares PEAT with CondSeg (Lei et al., 2025), DS-TransUNet-B (Lin et al., 2022), MPEDA-Net (Luo et al., 2024), NormalNet (Liu H. et al., 2024), Rolling-UNet-S (Liu Y. et al., 2024), SEDNet (Olisah and Van Cauter, 2024), TransUNet (Chen et al., 2021), U-KAN (Li et al., 2025), UNeXt (Valanarasu and Patel, 2022), and SegMamba (Xing et al., 2024). The available comparison records use the same test split. The main tables report Accuracy, F-score, Precision, Dice, parameter count, and inference throughput. For every case, the kidney and tumor ground-truth masks are combined, and their minimum 3D bounding box is computed. Each of the six box boundaries (lower and upper bounds on the *z, y*, and *x* axes) is expanded by 50 voxels and clamped to the image boundary. The same ROI extraction procedure is applied to all methods, after which 2D slices are resized to 256 × 256, providing consistent offline evaluation inputs for all compared methods.

### 4.2 Parameter settings

All input slices are resized to 256 × 256. Training augmentation includes random horizontal flipping, vertical mirroring, and random rotation. The reported configuration uses 16 Transformer blocks with patch size 16; BRA has *L* = 8 layers, *n*_win_ = 7 window partitions per spatial axis, and Top-*k* routing parameter *k* = 4. The output has three classes (background, kidney, and kidney tumor). Cross-entropy and Dice losses are jointly optimized with equal weights, and the BSA alignment-loss weight is set to λ = 1, using Adam with an initial learning rate of 1 × 10^−3^, polynomial decay (power 0.9; minimum 1 × 10^−5^), and 20,000 iterations. During training, frozen MedSAM image-encoder embeddings provide the BSA feature reference. The MedSAM branch is removed from the inference pathway. Experiments were run on Ubuntu 20.04 with Python 3.8, PyTorch 1.10.0, and CUDA 11.3, using a server equipped with two NVIDIA RTX 3090 GPUs (24 GB each), 40 vCPUs on an AMD EPYC 7642 platform, and 180 GB system memory. Mixed precision was not used. Training used both GPUs, with the batch size adjusted separately for each model. The FPS benchmark used one RTX 3090 for every 2D model, fixed 256 × 256 inputs, batch size 8, and 1,000 test images (125 batches). Average throughput was calculated as 1,000 divided by total inference time. Because SegMamba uses native 3D case-level execution, its FPS entry is shown as a dash.

### 4.3 Quantitative comparison and analysis

This study adopts Accuracy, F-score, Precision, and Dice as the evaluation metrics. These overlap-oriented measures are widely used for kidney and tumor segmentation and characterize segmentation quality from complementary perspectives (Maier-Hein et al., 2024). Tables 1–4 report these four metrics, together with parameter count and inference throughput.

**Table 1:** Quantitative comparison of kidney segmentation performance on KiTS21.

Algorithm	Accuracy	F-score	Precision	Dice	Params (M)	FPS
CondSeg	93.91	95.84	96.24	96.43	45.6	8.40
DS-TransUNet-B	88.41	92.06	92.83	93.20	171.5	10.50
MPEDA-Net	94.48	95.98	96.11	96.56	0.9	37.20
NormalNet	92.97	95.43	96.16	96.09	58.2	12.50
Rolling-UNet-S	94.15	95.92	96.22	96.50	1.8	77.20
SEDNet	93.15	95.45	96.05	96.10	1.3	87.70
TransUNet	91.05	94.88	**96.57**	95.61	105.3	7.20
U-KAN	92.96	95.59	96.50	96.22	6.4	63.30
UNeXt	94.15	95.65	95.68	96.27	1.5	103.00
SegMamba	94.45	95.72	96.31	96.27	67.4	–
PEAT	**94.69**	**96.43**	96.40	**97.69**	139.8	21.11

Accuracy, F-score, Precision, and Dice are reported as percentages; bold indicates the highest value and underlining indicates the second-highest. Params are in millions, with the training-stage MedSAM branch excluded from the PEAT inference pathway. FPS is measured on one RTX 3090 with fixed 256 × 256 inputs and 1,000 test images (Section 4.2); because each method's network architecture and input size are identical across the four tasks, its Params and FPS values are likewise identical across tasks. The SegMamba FPS entry is shown as a dash because its native 3D execution is not directly comparable.

Table 1 reports the KiTS21 kidney results. PEAT obtains the highest Accuracy (94.69), F-score (96.43), and Dice (97.69) among the listed methods. Relative to the second-highest values, these scores are higher by 0.21, 0.45, and 1.13 percentage points, respectively. TransUNet records the highest Precision (96.57), followed by U-KAN (96.50), while PEAT reaches 96.40. The combination of the highest Dice and F-score indicates that PEAT improves overall overlap without relying solely on the Precision column.

Table 2 presents the more difficult KiTS21 tumor task. PEAT achieves the highest F-score (90.46), Precision (90.28), and Dice (91.83). Its Dice is 0.64 percentage points above MPEDA-Net (91.19), and its Precision is 2.44 points above SegMamba (87.84), the second-highest entry in that column. U-KAN has the highest Accuracy (90.56), with PEAT second at 90.53.

**Table 2:** Quantitative comparison of tumor segmentation performance on KiTS21 under the same Params/FPS protocol as Table 1.

Algorithm	Accuracy	F-score	Precision	Dice	Params (M)	FPS
CondSeg	87.15	88.42	86.63	90.08	45.6	8.40
DS-TransUNet-B	74.83	76.11	72.29	79.54	171.5	10.50
MPEDA-Net	89.74	89.72	87.39	91.19	0.9	37.20
NormalNet	79.79	76.89	71.52	80.20	58.2	12.50
Rolling-UNet-S	87.79	89.00	87.30	90.58	1.8	77.20
SEDNet	87.06	88.82	87.43	90.42	1.3	87.70
TransUNet	87.55	86.08	82.31	88.07	105.3	7.20
U-KAN	**90.56**	88.78	85.29	90.39	6.4	63.30
UNeXt	88.60	89.16	87.09	90.72	1.5	103.00
SegMamba	90.01	89.89	87.84	90.92	67.4	–
PEAT	90.53	**90.46**	**90.28**	**91.83**	139.8	21.11

All segmentation metrics are percentages; bold indicates the highest value and underlining indicates the second-highest.

For KiTS23 kidney segmentation (Table 3), PEAT again has the highest Accuracy (93.39), F-score (95.09), and Dice (96.80). The corresponding margins over the second-highest results are 0.71, 0.19, and 0.73 percentage points. U-KAN yields the highest Precision (96.34), while PEAT records 95.14. Thus, the KiTS23 kidney results reproduce the main KiTS21 trend: PEAT leads the overlap-oriented Dice and F-score measures, whereas another method attains the highest Precision.

**Table 3:** Quantitative comparison of kidney segmentation performance on KiTS23 under the same Params/FPS protocol as Table 1.

Algorithm	Accuracy	F-score	Precision	Dice	Params (M)	FPS
CondSeg	90.97	94.42	95.67	95.22	45.6	8.40
DS-TransUNet-B	85.35	89.70	90.40	91.18	171.5	10.50
MPEDA-Net	92.35	94.69	95.12	95.45	0.9	37.20
NormalNet	90.75	93.01	92.96	94.01	58.2	12.50
Rolling-UNet-S	91.91	94.90	95.91	95.63	1.8	77.20
SEDNet	91.94	94.60	95.25	95.37	1.3	87.70
TransUNet	89.33	93.02	94.09	94.02	105.3	7.20
U-KAN	90.65	94.61	**96.34**	95.38	6.4	63.30
UNeXt	92.39	94.81	95.33	95.55	1.5	103.00
SegMamba	92.68	94.78	95.58	96.07	67.4	–
PEAT	**93.39**	**95.09**	95.14	**96.80**	139.8	21.11

All segmentation metrics are percentages; bold indicates the highest value and underlining indicates the second-highest.

Table 4 shows the KiTS23 tumor results. PEAT reaches the highest F-score (90.57), Precision (88.72), and Dice (92.58). The Dice improvement over SegMamba, the second-highest method, is 0.69 percentage points. U-KAN reports the highest Accuracy (92.79), followed by UNeXt (92.58); PEAT is close at 92.54.

**Table 4:** Quantitative comparison of tumor segmentation performance on KiTS23 under the same Params/FPS protocol as Table 1.

Algorithm	Accuracy	F-score	Precision	Dice	Params (M)	FPS
CondSeg	86.35	82.64	77.43	85.13	45.6	8.40
DS-TransUNet-B	76.20	78.00	74.66	81.15	171.5	10.50
MPEDA-Net	88.47	89.18	87.19	90.73	0.9	37.20
NormalNet	74.34	76.89	73.75	80.20	58.2	12.50
Rolling-UNet-S	92.13	89.97	86.47	91.41	1.8	77.20
SEDNet	89.99	90.35	88.38	91.73	1.3	87.70
TransUNet	85.20	86.95	85.17	88.82	105.3	7.20
U-KAN	**92.79**	89.60	85.50	91.09	6.4	63.30
UNeXt	92.58	90.41	86.98	91.79	1.5	103.00
SegMamba	92.41	90.28	88.01	91.89	67.4	–
PEAT	92.54	**90.57**	**88.72**	**92.58**	139.8	21.11

All segmentation metrics are percentages; bold indicates the highest value and underlining indicates the second-highest.

In both tumor tasks, PEAT outperforms all compared methods in both Dice and Precision, demonstrating its advantage on the smaller and more heterogeneous target class.

Because each method's network architecture and input size are identical across the four tasks, the Params and FPS values are the same in Tables 1–4. Under the benchmark protocol described in Section 4.2, the inference-time PEAT task network contains 139.8 M parameters and processes 21.11 images/s, with the frozen MedSAM branch absent from this pathway. Its throughput is higher than TransUNet, CondSeg, DS-TransUNet-B, and NormalNet, and lower than MPEDA-Net, U-KAN, Rolling-UNet-S, SEDNet, and UNeXt. PEAT has the second-largest parameter count among the listed task networks, after DS-TransUNet-B.

### 4.4 Qualitative comparison and analysis

Figures 4, 5 show representative qualitative comparisons on KiTS21 and KiTS23. Each row is one selected sample; columns show the reference mask, PEAT, and the listed baselines. Red indicates tumor predictions and green indicates kidney predictions.

**Figure 4:**
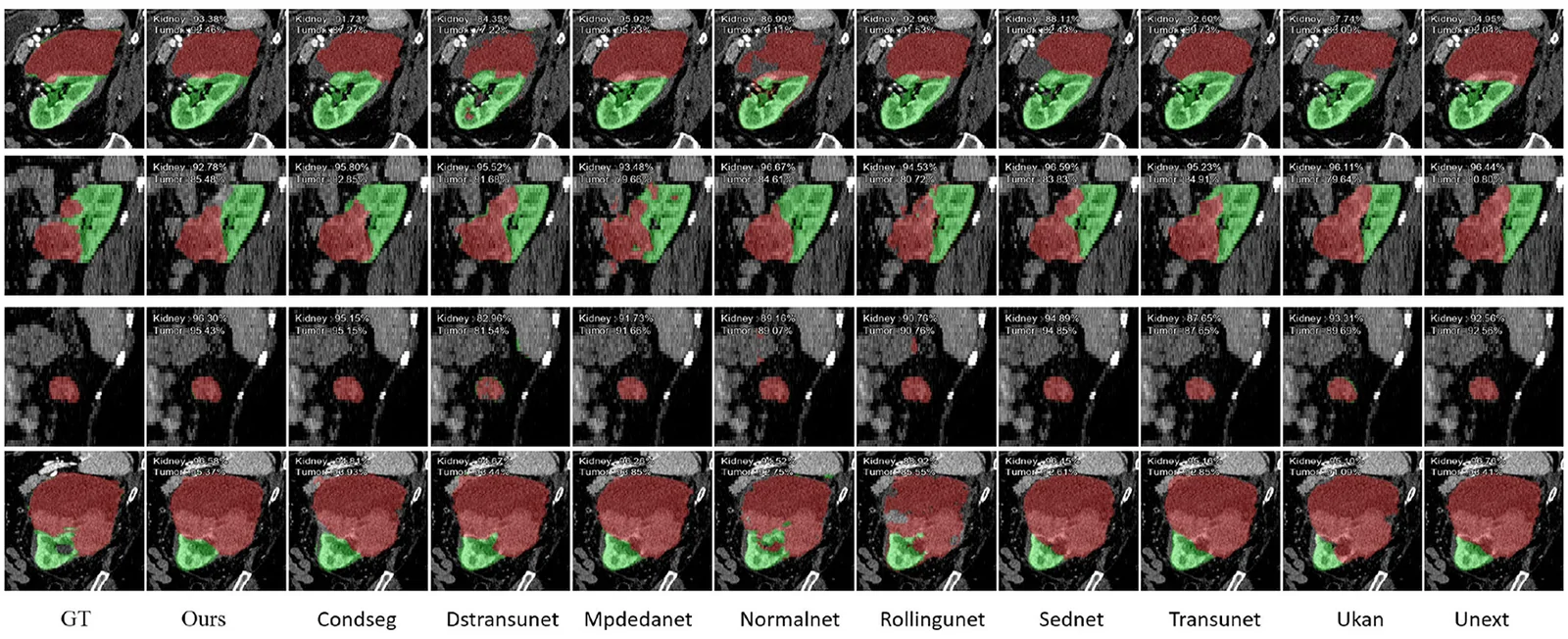
Qualitative comparison on KiTS21. Each row presents a representative test slice, and columns show the ground truth (GT), PEAT (Ours), and the listed comparison methods. Green and red overlays denote kidney and tumor regions, respectively.

**Figure 5:**
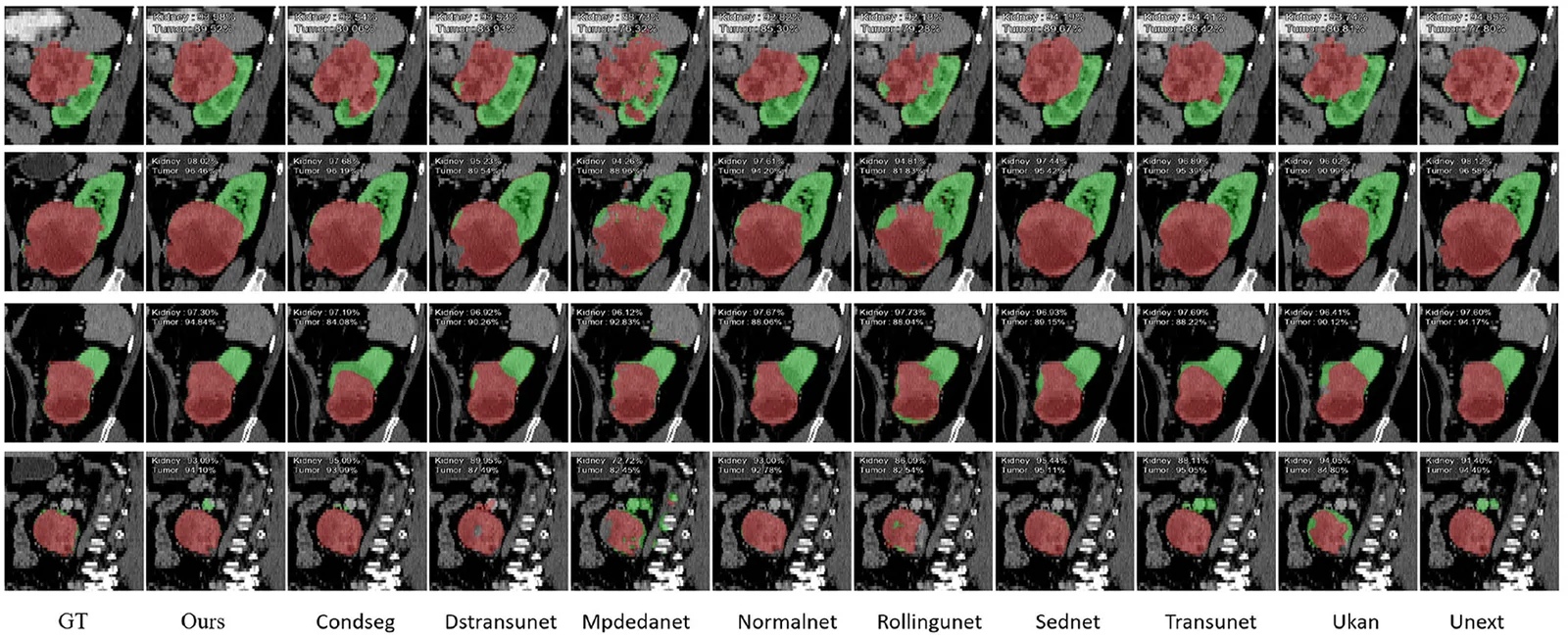
Qualitative comparison on KiTS23. Each row presents a representative test slice, and columns show the ground truth (GT), PEAT (Ours), and the listed comparison methods. Green and red overlays denote kidney and tumor regions, respectively.

Figure 4 includes four cases with different tumor sizes and tumor–kidney relationships. In the first and fourth rows, the tumor occupies a large part of the visible renal region. PEAT produces a continuous tumor mask and retains the adjacent kidney region, whereas several comparison outputs contain visible internal gaps, irregular protrusions, or incomplete separation at the interface. The second row contains an exophytic mass with a more complex contact surface; PEAT follows the main reference extent while some alternatives introduce small isolated regions or local leakage. In the third row, the lesion is compact and occupies a much smaller image area. PEAT retains a single connected response close to the reference, while several baselines visibly under-segment or fragment the lesion. These examples illustrate the different demands posed by large masses and small focal lesions.

Figure 5 presents another four cases with irregular boundaries, large exophytic tumors, compact lesions, and small separated regions. In the first row, the tumor–kidney interface is highly irregular; PEAT preserves the principal tumor extent without the conspicuous internal omissions visible in several alternative predictions. In the second row, PEAT produces a contiguous mask for the large tumor and maintains the neighboring kidney component. The third row demonstrates that the model also retains a compact lesion with a smooth main contour. In the final row, where the target occupies a small and visually difficult region, PEAT captures the principal lesion and the small adjacent kidney component shown in the reference. The visual observations agree with the leading Dice values in Tables 3, 4.

The figures illustrate where different methods add, omit, or fragment regions in the selected samples and provide a visual counterpart to the overlap-based comparisons.

Figure 6 shows Grad-CAM visualizations for selected KiTS21 and KiTS23 examples. The strongest activation is concentrated over the renal or tumor-containing region in each displayed slice, while the response decreases toward much of the surrounding background. Activation also extends around portions of the target boundary, indicating that the prediction uses both the interior appearance and neighboring context. These heatmaps provide an interpretable view of the regions emphasized by the network in the selected cases.

**Figure 6:**
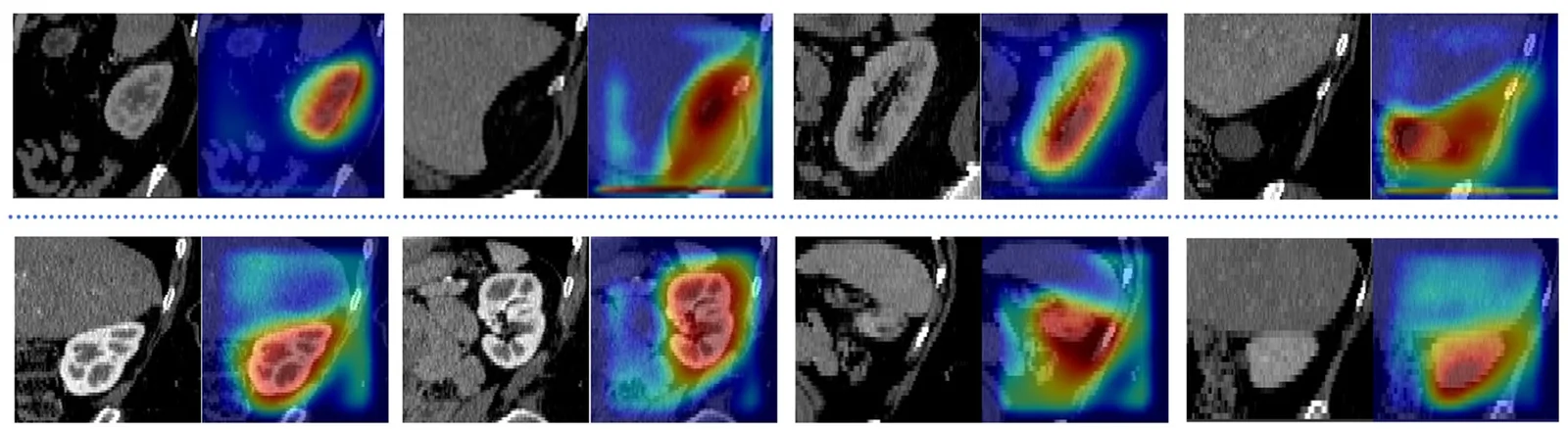
Activation heatmaps of PEAT on representative KiTS21 and KiTS23 slices. Each grayscale CT image is paired with its activation map; warmer colors indicate stronger model responses in the kidney or tumor region.

### 4.5 Ablation

Tables 5, 6 evaluate the BSA distance function and the contribution of each architectural component. Table 5 compares L1, L2, and cosine alignment under the matched training protocol. L1 has the highest Dice on all four tasks. The differences from the strongest alternative are 0.18 points for KiTS21 kidney, 0.21 for KiTS21 tumor, 0.14 for KiTS23 kidney, and 0.13 for KiTS23 tumor. The close results indicate that BSA is not highly sensitive to the specific distance form, although L1 is consistently the best of the three tested choices.

**Table 5:** Alignment-loss Dice ablation under the matched training protocol.

Loss	KiTS21 kidney	KiTS21 tumor	KiTS23 kidney	KiTS23 tumor
L1	**97.69**	**91.83**	**96.80**	**92.58**
L2	97.51	91.45	96.62	92.45
Cosine	97.48	91.62	96.66	92.36

All Dice values are percentages; bold indicates the highest value and underlining indicates the second-highest.

**Table 6:** Module-combination Dice ablation.

Configuration	KiTS21 kidney	KiTS21 tumor	KiTS23 kidney	KiTS23 tumor
Baseline	95.61	88.07	94.02	88.82
BSA	96.17	88.64	94.71	90.80
DAF	96.22	88.75	95.16	89.74
BRA	95.87	89.17	95.45	91.16
BSA+DAF	96.93	90.55	95.89	91.92
BSA+BRA	96.78	90.57	95.97	91.87
DAF+BRA	97.21	91.28	96.42	92.15
PEAT	**97.69**	**91.83**	**96.80**	**92.58**

All Dice values are percentages; bold indicates the best value and underlining indicates the strongest pairwise configuration.

Table 6 first examines the individual modules. Relative to the baseline, BSA improves Dice by 0.56, 0.57, 0.69, and 1.98 percentage points on KiTS21 kidney, KiTS21 tumor, KiTS23 kidney, and KiTS23 tumor, respectively. DAF produces gains of 0.61, 0.68, 1.14, and 0.92 points, showing consistent improvement from multi-scale local–contextual fusion. BRA improves the same tasks by 0.26, 1.10, 1.43, and 2.34 points. The larger BRA gains on the tumor tasks, especially KiTS23, are consistent with the value of routed contextual interaction for heterogeneous lesions.

The pairwise configurations provide a direct test of module stacking. BSA+DAF reaches 96.93, 90.55, 95.89, and 91.92 Dice; BSA+BRA reaches 96.78, 90.57, 95.97, and 91.87; and DAF+BRA reaches 97.21, 91.28, 96.42, and 92.15. Every pairwise result exceeds the stronger of its two corresponding single-module results on every task, with improvements ranging from 0.52 to 2.11 percentage points. This demonstrates that combining the modules improves performance rather than reducing the benefit of either component.

DAF+BRA is the strongest pairwise configuration across all four tasks. Adding BSA to form the complete PEAT model further increases Dice by 0.48, 0.55, 0.38, and 0.43 percentage points, respectively. Relative to the baseline, full PEAT improves Dice by 2.08 points on KiTS21 kidney, 3.76 on KiTS21 tumor, 2.78 on KiTS23 kidney, and 3.76 on KiTS23 tumor. These results demonstrate that the progressive integration of semantic alignment, multi-scale fusion, and routed region interaction yields the best configuration in every evaluated task.

## 5 Discussion

On the two KiTS benchmarks, the complete PEAT configuration achieves the highest Dice among the compared methods in all four kidney and tumor segmentation tasks and also leads F-score in each task, while the highest Accuracy or Precision is occasionally obtained by another method. This pattern indicates that the improvement of PEAT is expressed primarily in overall segmentation overlap and agreement rather than in uniform dominance of every metric.

The ablation results further clarify the complementary roles of the three components: MedSAM provides a broad medical feature reference while the PEAT task pathway remains specialized through direct KiTS supervision; the feature alignment of BSA is effective under three common distance measures, with L1 giving a small and consistent advantage; DAF performs local–global feature integration at multiple encoder levels; and BRA performs content-routed cross-region interaction at the bottleneck. Every pairwise configuration outperforms its constituent single modules, and the complete model further outperforms the strongest pairwise configuration (DAF+BRA), supporting the design hypothesis that the three components act at different stages of representation learning and that their gains are additive.

The computational overhead places these accuracy results in context. The inference-time PEAT task network reaches 21.11 FPS on one RTX 3090, which is faster than TransUNet, CondSeg, DS-TransUNet-B, and NormalNet but slower than several compact CNN-oriented models; its 139.8 M parameters are fewer than DS-TransUNet-B and more than the other listed methods. Overall, PEAT trades model size and inference overhead for higher segmentation accuracy and richer combined representation capacity.

This study has limitations. PEAT operates on 2D axial slices and does not explicitly model the 3D anatomical continuity across slices, so the volumetric spatial context of the kidney and tumors is not fully exploited. Future work will focus on extending PEAT to 3D volumetric segmentation to further exploit cross-slice anatomical priors and improve overall segmentation consistency.

## 6 Conclusion

This paper presented PEAT, a kidney and renal-tumor segmentation framework that combines foundation-model priors with task-specific learning: BSA aligns the bottleneck representation with the frozen MedSAM image embedding through an L1 constraint during training; DAF fuses CNN local features with MaxViT contextual features at multiple encoder scales by cascading coordinate attention and SENetV2; and BRA models non-local dependencies at the bottleneck with Top-*k* window routing and patch-level sparse interaction. The task network is supervised directly by KiTS annotations and performs inference independently.

On the KiTS21 and KiTS23 benchmarks, PEAT achieves kidney and tumor Dice scores of 97.69%/91.83% and 96.80%/92.58%, respectively, all the highest among the compared methods; the ablation studies show that the integration of the three components yields consistent and additive gains, with the complete model outperforming all single and pairwise configurations. These results indicate that combining training-stage broad feature guidance with task-specific representation learning is an effective route to improving kidney and renal-tumor segmentation, and may inform the coordinated design of foundation-model priors and task networks.

## Data Availability

The raw data supporting the conclusions of this article will be made available by the authors, without undue reservation.
